# Influencing factors of futile recanalization after endovascular therapy for cerebral infarction with posterior circulation occlusion of large vessels: a retrospective study

**DOI:** 10.1186/s12883-023-03166-x

**Published:** 2023-03-29

**Authors:** Ying Xie, Xi Liu, Hongli Gu, Guanghong Zhong, Yangchun Wen, Jinzhao He, Xiaojin Zhong

**Affiliations:** Department of Neurology, Heyuan People’s Hospital, Heyuan, 517000 Guangdong China

**Keywords:** Cerebral infarction, Endovascular therapy, Futile recanalization, Posterior circulation occlusion

## Abstract

**Background:**

The optimal treatment for cerebral infarction caused by posterior circulation occlusion of large vessels has not yet been determined. Intravascular interventional therapy is an important treatment for cerebral infarction with posterior circulation occlusion of large vessels. However, endovascular therapy (EVT) of some posterior circulation cerebrovascular is ineffective and eventually become futile recanalization. Therefore, we performed a retrospective study to explore the factors influencing futile recanalization after EVT in patients with posterior circulation large-vessel occlusion.

**Methods:**

Eighty-six patients with acute cerebral infarction and posterior circulation large vessel occlusion after intravascular intervention were divided into two groups according to their modified Rankin scale (mRS) scores after 3 months: group 1, mRS scores less than or equal to 3 (the effective recanalization group); group 2, mRS scores greater than 3 (the ineffective recanalization group). The basic clinical data, imaging index scores, time from onset to recanalization, and operation time between the two groups were compared and analyzed. Logistic regression was used to analyze the factors influencing indicators of good prognosis, and the ROC curve and Youden index were used to determine the best cutoff value.

**Results:**

Between the two groups, there were significant differences in the posterior circulation CT angiography (pc-CTA) scores, GCS scores, pontine midbrain index scores, time from discovery to recanalization, operation time, NIHSS score and incidence of gastrointestinal bleeding. The logistic regression revealed that the NIHSS score and time from discovery to recanalization were associated with good prognoses.

**Conclusion:**

NIHSS score and recanalization time were independent influencing factors of ineffective recanalization of cerebral infarctions caused by posterior circulation occlusion. EVT is relatively effective for cerebral infarction caused by posterior circulation occlusion when the NIHSS score is less than or equal to 16 and the time from onset to recanalization is less than or equal to 570 min.

**Supplementary Information:**

The online version contains supplementary material available at 10.1186/s12883-023-03166-x.

## Introduction

Posterior circulation cerebral infarction accounts for approximately 20% of ischemic stroke cases [1]. One in 20 cases with posterior circulation cerebral infarction occurs due to occlusion of large vessels [2]. Cerebral infarction caused by occlusion of posterior circulation large vessels has a high mortality and disability rate. For example, the death rate of cerebral infarction due to basilar artery occlusion can be as high as 80% [3, 4]. Previous studies have shown that endovascular therapy (EVT) is effective and safe for cerebral infarction caused by occlusion of large vessels, including in the anterior circulation and post circulation [5–7]. However, some patients with cerebral infarction with posterior circulation occlusion have poor results after EVT. What’s more, some patients with large posterior circulation occlusion who received interventional vessel opening eventually became futile recanalization [8].

Futile recanalization is defined by endovascular treatment to recanalize an occluded vessel and restore the patient’s blood flow to a modified treatment in cerebral ischemia (mTICI) score of grade 2b or 3, after which the patient does not achieve functional independence [9, 10]. Compared with anterior circulation cerebral infarction, mRS Score (Modified Rankin Scale) less than or equal to 3 in posterior circulation cerebral infarction is usually regarded as the outcome index of good function [6, 7]. The pathological mechanism of futile recanalization remains unclear and may be related to reperfusion injury and distal microvascular embolism. The reason why the probability of futile recanalization of cerebral infarction in the posterior circulation is higher than that in the anterior circulation may be that the collateral circulation in the posterior circulation is relatively less extensive [11, 12].

Although a few studies have found that futile recanalization of posterior circulation large-vessel occlusion is affected by multiple factors [13], these have yet to be fully clarified. In clinical practice, whether EVT should be applied for the treatment of posterior circulation occlusion of large vessels remains unclear. Therefore, we performed a retrospective study to explore the factors influencing futile recanalization after EVT in patients with posterior circulation large-vessel occlusion.

## Materials and methods

### Patients

**Fig. 1:**
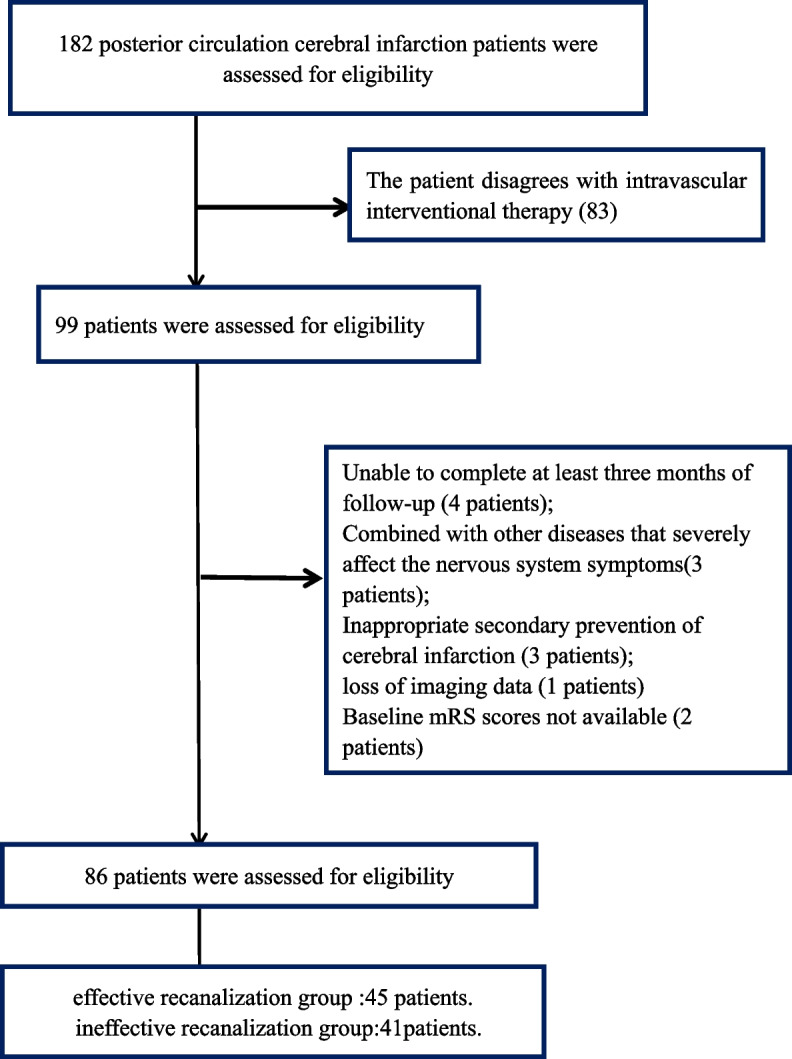
The enrollment diagram for posterior circulation cerebral infarction patients

All data in this retrospective study were collected from the Heyuan People’s Hospital from 2018 to 2022. The inclusion criteria were as follows: 1) age ≥ 18 years; 2) occlusion of the vertebral, basilar, or posterior cerebral artery confirmed by baseline computed tomography angiography, with symptoms attributable to ischemia of the posterior circulation; and 3) patients achieving effective recanalization (reperfusion was defined as eTICI 2B-3). The exclusion criteria were as follows: 1) unavailable baseline modified Rankin scale (mRS) scores; 2) inability to complete at least 3 months of follow-up; 3) patients with a combination of other diseases that severely affect the nervous system; 4) inappropriate secondary prevention of cerebral infarction during follow-up; and 5) loss of imaging data (Fig. 1).

### Treatment procedures

The endovascular interventions included digital subtraction angiography followed by or without mechanical thrombectomy. The mechanical thrombectomy methods included stent thrombectomy, thrombus aspiration, stenting, or a combination of modalities with or without the delivery of thrombolytic agents. EVT was performed at the discretion of the attending physician. The preferred method of anesthesia was local anesthesia, general anesthesia, or a combination of both, and the method was determined according to the specific individual characteristics of the patient. All patients received standard care during their hospitalization and follow-up.

### Imaging assessments

Two blinded, independent, and experienced neuroradiologists performed all imaging assessments. MRI pc-ASPECTS, pc-CTA score, pontine-midbrain index score and postoperative vascular occlusion were assessed. The pc-ASPECTS score divides the posterior circulation into 10 points minus 1 point for each infarcted area of the lesion in the left and right thalamus, cerebellum, and posterior cerebral arteries, and 2 points for any affected region in the midbrain or pons. Pc-CTA scores were assessed according to the scoring methods based on the DSA results described in the references [14]. A score of 0 indicated no vascular occlusion, and a score of 6 indicated complete occlusion of the posterior circulation, including at least one vertebral artery and the bilateral posterior cerebral arteries. The ponto-midbrain index was also assessed based on the literature [15]. Based on cranial MR images, 0 indicated no hyperintense infarct areas, 1 indicated a hyperdense area less than 50%, 2 indicated a hyperdense area > 50%, and the pons and midbrain were divided into left and right locations for a total of four locations for scoring.

The assessment of postoperative responsible vessel occlusion was based on postoperative CTA or MRA.

### Time metrics

All-time variables were assessed using a standardized approach consistent with the definitions used in previous studies on basilar artery strokes. The time of first symptom onset was reported as observed if the onset was witnessed or as the time last known well if the onset was not witnessed. In patients with transient or mild neurological symptoms with secondary worsening consistent with posterior circulation cerebral infarction, the time point of secondary worsening was considered the estimated time of posterior circulation cerebral infarction.

### Outcome assessments

All patients were followed-up for 3 months, and the mRS scores in the acute phase were measured after treatment (at the 3-month follow-up). According to their 3-month mRS scores, the enrolled patients were divided into two groups: group 1, the effective recanalization group (mRS scores less than or equal to 3 points); group 2, the ineffective recanalization group (mRS scores greater than 3 points) [16]. Baseline data in the two groups, including age, sex, past history, pc-ASPECTS, pc-CTA, preoperative GCS score (Glasgow score), the National Institutes of Health Stroke Scale (NIHSS) score, pontine-midbrain index score, blood test results, time from onset to recanalization, operation time and postoperative complications were collected and included in the statistics.

### Statistical analysis

Statistical analysis was performed using SPSS 22.0, and the measurement data were expressed as the mean and standard deviation. Each data group was subjected to normality and homogeneity of variance tests, and the statistical method was chosen based on whether the data followed a normal distribution. Differences between two groups were measured using a paired rank-sum test. The rank-sum test with two independent samples and chi-square test were used to compare the differences between the two groups. In both groups, 3-month good prognosis (mRS) served as the statistical endpoint, and logistic regression was used to identify influential factors. Logistic regression was used for univariate analysis, and the influencing factors with *P* value less than 0.01 were included in the multivariate logistic regression analysis. The independent influencing factors of ineffective recanalization of posterior circulation cerebral infarction were obtained. The sensitivity and specificity values were calculated using an ROC curve and the Youden index, and the statistical significance was set at *p* < 0.05. X-tile software is used to calculate cut off value of independent influencing factors.

The ethics committee of Heyuan People’s Hospital approved the retrospective analysis of patient data.

## Results

### Clinical features

After excluding 96 patients who failed to meet the study criteria, 86 were included in our analysis (Fig. 1).

The effective recanalization group comprised 45 patients, including 10 women and 35 men, with an average age of 62.3 years. In this group, the preoperative pc-ASPECTS score was 7 points. The preoperative GCS score was 9 points. The preoperative NIHSS score was 16 points. The time from onset to recanalization was 545 min. The operation time was 107 min. The preoperative pc-CTA score was 2 points. The preoperative midbrain-pontine index score was 1 point. Thirty-five patients developed pulmonary infections. Postoperative gastrointestinal bleeding occurred in 3 patients. One patient was found vascular reocclusion, and no symptomatic cerebral hemorrhage or brain herniation was found in the included patients.

The ineffective recanalization group comprised 41 patients, including 8 women and 33 men, with an average age of 65.5 years. In this group, the preoperative pc-ASPECTS score was 6 points. The preoperative GCS score was 6 points. The preoperative NIHSS score was 27 points. The time from onset to recanalization was 859 min. The operation time was 136 min. The preoperative pc-CTA score was 3 points. The preoperative midbrain-pontine index score was 2 points. Thirty-six patients developed pulmonary infections. Postoperative gastrointestinal bleeding occurred in 17 patients. Six patients were found vascular reocclusion.

**Fig. 2:**
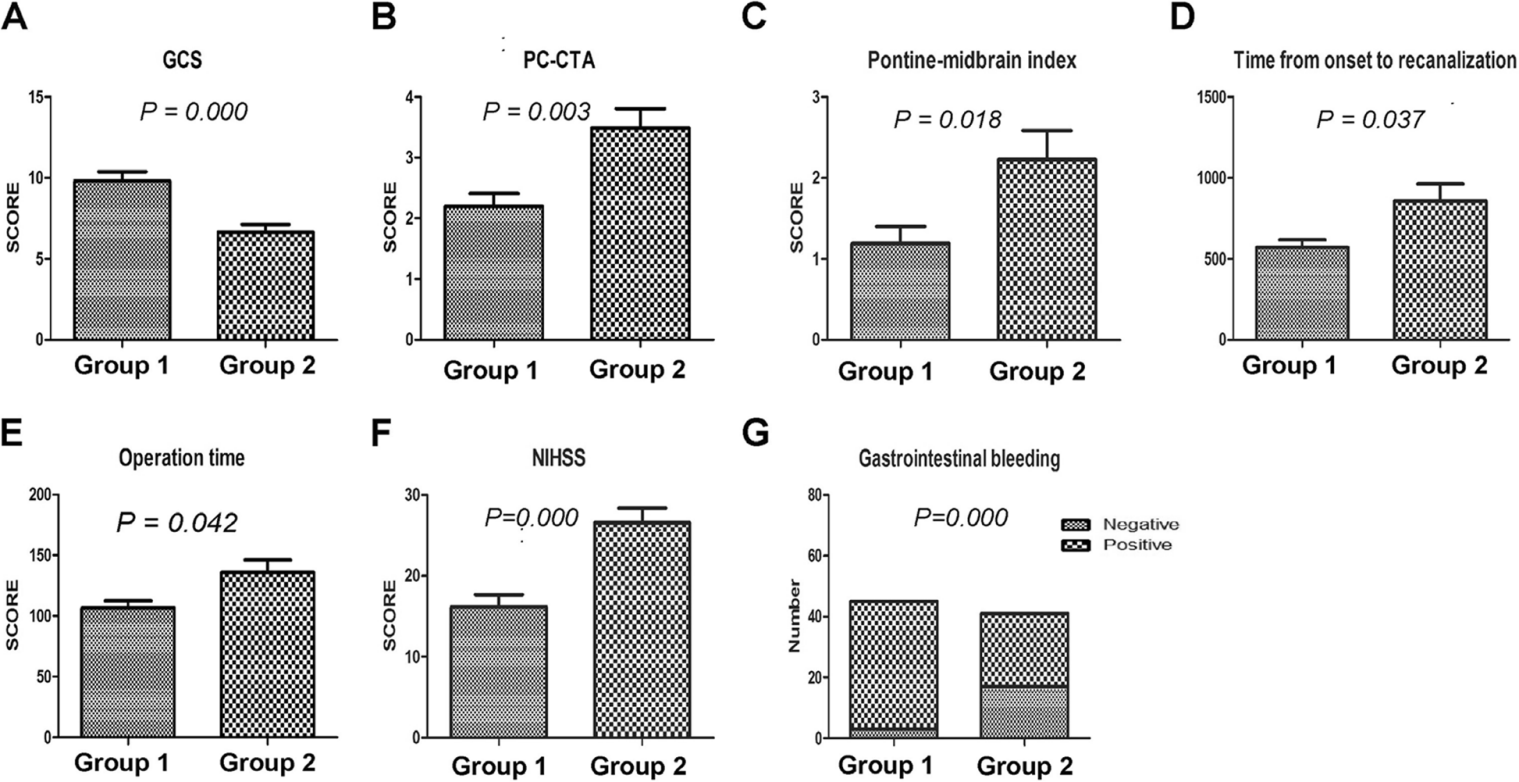
The different clinical characteristics between two groups. Legends: Group1: the effective recanalization group. Group2: the ineffective recanalization group. There were statistical differences between two groups in GCS score(**A**), PC-CTA score(**B**), pontine midbrain index score(**C**), time from onset to vascular recanalization(**D**), operation time(**E**), NIHSS score(**F**) and Gastrointestinal bleeding(**G**)

Between the two groups, there were significant differences in the pc-CTA scores (*p* = 0.003), GCS scores (*p* = 0.000), NIHSS score (*p* = 0.000), pontine midbrain index scores (*p* = 0.018), time from discovery to recanalization (*p* = 0.037), operation time (*p* = 0.042) and gastrointestinal bleeding (*p* = 0.000) (Fig. 2). There were no significant differences between the two groups in terms of sex, age of onset, history of hypertension, history of diabetes, intravenous thrombolysis, history of smoking, history of drinking, preoperative serological leukocytes, PC-ASPECTS scores, atrial fibrillation, preoperative blood glucose levels and other postoperative complications (Table 1) (Supplementary 1).

**Table 1:** Differences in baseline clinical characteristics between the two groups

	**Effective recanalization group**	**Ineffective recanalization group**	***P* value**
**Patients, NO**	45	41	
**Gender**			0.758
** Male**	35	33	
** Female**	10	8	
**Age, mean(years)**	62.3 + 13.4	65.3 + 12.0	0.892
**Hypertension**	15	13	0.872
**Diabetes**	29	29	0.534
**Hyperlipidemia**	33	35	0.171
**Atrial fibrillation**	8	2	0.093
**Coronary heart disease**	1	4	0.126
**Previous stroke**	2	2	0.924
**Drinking**	31	32	0.338
**Smoking**	24	25	0.475
**TOAST etiological classification**			0.591
large artery atherosclerosis	30	29	
cardiogenic embolism	9	5	
unknown cause	6	7	
**Intravenous thrombolysis**	14	16	0.442
**White blood cell count**	10.13 + 3.5	10.97 + 3.9	0.354
**Preoperative blood glucose**	9.8 + 4.5	9.8 + 4.1	0.677
**NIHSS score**	16 + 10	27 + 11	0.000
**PC-CTA score**	2 + 1	3 + 2	0.003
**GCS score**	9 + 4	6 + 3	0.000
**PC-aspects score**	7 + 2	6 + 2	0.368
**Midbrain-pontine index score**	1 + 1	2 + 2	0.018
**Time from discovery to recanalization**	545 + 250	859 + 675	0.037
**Operation time**	107 + 40	136 + 67	0.042
**Postoperative complications**			
**Symptomatic cerebral hemorrhage**	0/45	4/41	-
**asymptomatic intracerebral hemorrhage**	2/45	3/41	0.666
**pulmonary infection**	35/45	36/41	0.221
**gastrointestinal bleeding**	3/45	17/41	0.000
**vascular reocclusion**	1/36	6/38	0.170

### The prognostic predictor in patients

Single factor analysis shows that invalid recanalization is related to time from discovery to recanalization, NIHSS score and Pons-Midbrain index. Multivariate logistic regression analysis showed that NIHSS score and time from discovery to recanalization were independent influencing factor of invalid recanalization (Table 2).

**Table 2:** Analyses of factors affecting prognosis of patients by logistric regression model

Clinical characteristics	Univariate analysis			Multivariate analysis		
	**OR**	**95% IC**	***P* value**	**OR**	**95% IC**	***P* value**
PC-CTA score	1.178	0.733–1.892	0.498			
Time from onset to recanalization	1.003	1.000–1.007	0.057	1.003	1.000–1.006	0.048
NIHSS score	1.102	1.023–1.188	0.011	1.103	1.029–1.183	0.006
Gastrointestinal bleeding	3.574	0.481–26.550	0.213			
Pons-Midbrain index	1.597	0.936–2.722	0.086	1.610	0.983–2.638	0.059
GCS score	1.035	0.763–1.404	0.826			
Operation time	1.016	0.996–1.037	0.122			

### The prognosis prediction efficiency in patients receiving endovascular interventional therapy

**Fig. 3:**
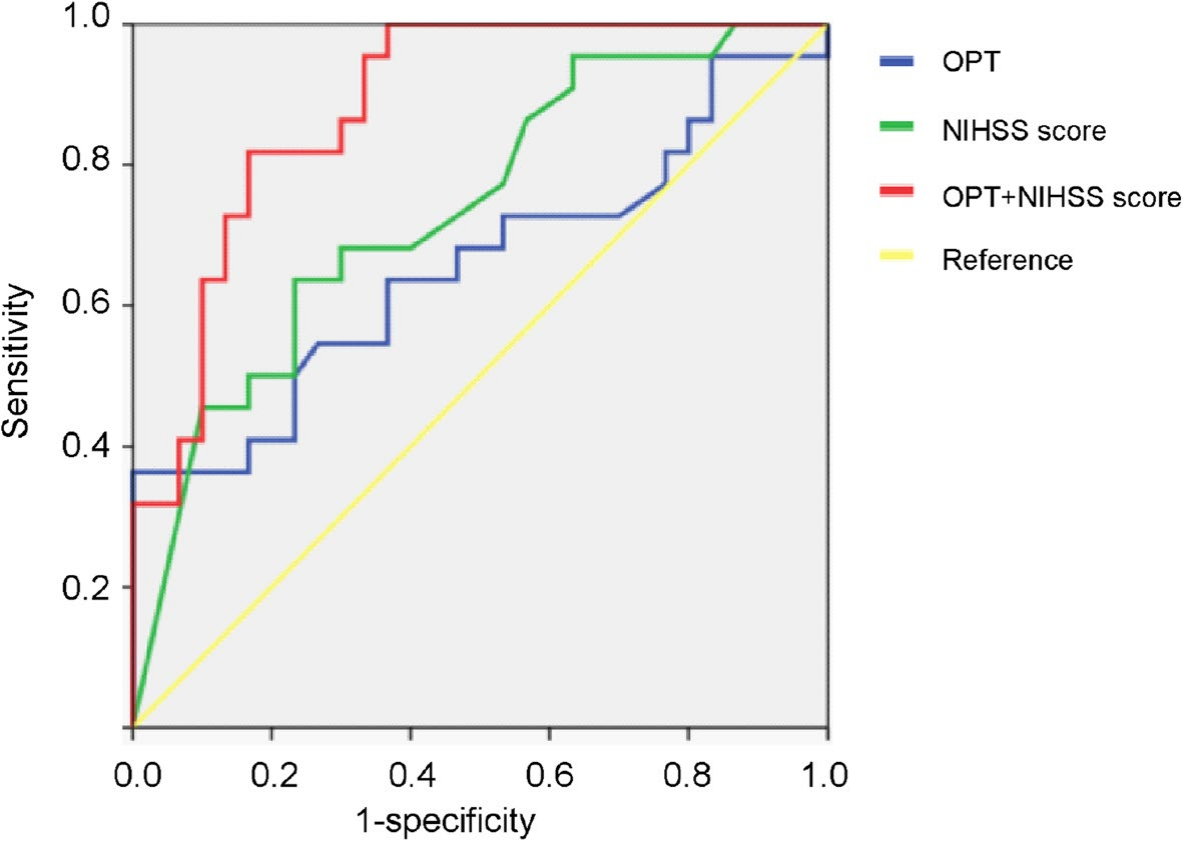
Ability of a combination of predictors to distinguish futile recanalization

The AUC of the NIHSS score for futile recanalization prediction efficiency in patients receiving endovascular interventional therapy was 0.737 the sensitivity was 0.636, and the specificity was 0.767, while the AUC of time from discovery to recanalization was 0.655, the sensitivity was 0.545 and the specificity was 0.733. The AUC of the NIHSS score plus time from discovery to recanalization was as high as 0.883, the sensitivity was 0.818, and the specificity was 0.833 (Fig. 3). Combined with the statistical results of this study and the needs of clinical practice, the best cutoff values may be NIHSS scores less than or equal to 16 and times from onset to recanalization no greater than 570 min.

## Discussion

Although more and more studies (BASLIAR [17], BAOCHE [6], ATTENTION [7, 18]) have demonstrated the effectiveness of intravascular intervention for acute posterior circulation large vessel occlusion in cerebral infarction. However, there are some large studies did not confirm that intravascular interventional therapy in the time window is superior to medical treatment alone for acute posterior circulation large-vessel occlusion stroke, including the BEST [19] and BASICS studies [20]. It is worth noting that the inclusion and exclusion criteria of the above studies were different, and different inclusion and exclusion criteria may lead to different and invalid recanalization rates, thus making the intravascular interventional treatment of posterior circulation large vessel occlusion cerebral infarction ambiguous in clinical practice [21]. Therefore, we consider that there are significant influencing factors for ineffective recanalization after EVT in cerebral infarction with posterior circulation large vessel occlusion.

Determining the influencing factors of ineffective recanalization following intravascular intervention will help to screen for cerebral infarction of posterior circulation large vessel occlusion cases that are suitable for intravascular intervention more accurately in clinical practice, which has great clinical practical significance. In our study, we assessed patients with cerebral infarction caused by posterior circulation great artery occlusion treated using interventional vascular therapy and compared the related characteristics of patients with effective recanalization and patients with ineffective recanalization. Our study found that NIHSS score and recanalization time were independent influencing factors for ineffective recanalization of cerebral infarction caused by posterior circulation occlusion of large arteries, with high specificity and sensitivity.

The recanalization time is the time from the onset of the patient to recanalization of the occluded vessel. Most previous studies have focused on the time of onset of cerebral infarction. The time window of EVT for cerebral infarction caused by posterior circulation occlusion was initially recommended to be 6 h; However, some guidelines now recommend that it be extended to 24 h [22, 23]. Other studies have focused on the timing of arterial puncture during vascular intervention. Although previous studies have shown that the optimal arterial puncture time is between 225 and 562 min [5, 24–26], the relevant timing is inconclusive. Considering that brain tissues that lack blood flow continue to die before the restoration of blood flow during EVT, we consider that patient prognoses may be affected by the time of onset and recanalization of EVT. Our study showed that the effective recanalization time was approximately 570 min. Except for the average operation time, the results were consistent with those of previous studies. Therefore, it is highly credible to consider recanalization time as an influencing factor in the efficacy of EVT for cerebral infarction caused by posterior circulation occlusion of large vessels.

NIHSS score is the most widely used scale in the world to assess the severity of stroke. It evaluates the severity, efficacy of treatment and prognosis of neurological impairment by scoring 11 neurological function indicators [27]. Previous studies have found that NIHSS score is of lower value in evaluating posterior circulation infarction than anterior circulation infarction. However, our study concluded that NIHSS score is an independent influencing factor for cerebral infarction caused by posterior circulation occlusion of the great arteries. When the NIHSS score was less than or equal to 16, the probability of effective recanalization was significantly increased during EVT. We speculate that the preoperative NIHSS score, as an index to evaluate the severity of patients, is closely related to the core area of the patients' posterior circulation infarction and the degree of vascular stenosis. Too high NIHSS score often represents that the core area of the brain stem has been seriously damaged, and this type of patients may be more prone to ineffective recanalization. However, in view of the limited value of NIHSS score for posterior circulation cerebral infarction, more adjusted or upgraded NIHSS score system is needed to describe posterior circulation cerebral infarction, and to evaluate the interventional therapy value and prognosis of posterior circulation cerebral infarction [28].

In addition to the above independent influencing factors, this study also found that there were statistically significant differences in the preoperative GCS scores, pc-CTA score, pontine-midbrain index scores, operation time and gastrointestinal bleeding between the two groups. The above influencing factors are similar to previous research results to varying degrees [29–32]. However, the logistic regression analysis revealed that these factors were not independent influencing factors. Because this was a single-center retrospective study, the evidence is relatively insufficient. We believe that research with a larger sample will further confirm the correlations among the preoperative GCS score, pc-CTA score, pontine-midbrain index scores, operation time and gastrointestinal bleeding with the effective recanalization of cerebral infarction caused by posterior circulation large vessel occlusion.

This study has the following limitations: (1) This study was a single-center study with a relatively small sample size; (2) the onset time of some patients was unclear: in this study, the last asymptomatic time was used as the onset time, which may have affected the recanalization time outcomes; (3) in this study, MR-DWI and T2-FLAIR sequence lesion matching was used as the surgical basis; thus, the lack of PWI, CTP, and other perfusion bases may have biased the outcomes; (4) this study is a retrospective study with a relatively low level of evidence and bias in some aspects, such as treatment methods and patient choice.

## Conclusion

NIHSS score and recanalization time were independent influencing factors for ineffective recanalization of cerebral infarction caused by posterior circulation occlusion. When the preoperative NIHSS scores of patients with cerebral infarction caused by posterior circulation occlusion are less than or equal to 16 and the time from onset to recanalization is less than or equal to 570 min, EVT will be relatively effective. EVT in such patients has a low probability of ineffective recanalization and good prognosis. In addition, preoperative GCS scores, pontine-midbrain index scores, pc-CTA score and gastrointestinal bleeding were potential factors influencing the ineffective recanalization of cerebral infarction caused by posterior circulation occlusion. However, further head-to-head randomized controlled trials are needed to verify the influencing factors derived from this study. We hope to identify more factors related to cerebral infarction caused by posterior circulation occlusion of the large vessels in the future, as these would provide powerful guidance for the clinical management of cerebral infarction caused by occlusion of the great arteries of the posterior circulation.

## Supplementary Information

**Additional file 1: Supplementary 1.** The baseline characteristics of patients in our study.

## Data Availability

The datasets used and/or analyzed during the current study are available from the corresponding author on reasonable request.

## Abbreviations

EVT: Endovascular therapy
mRS: Modified rankin scale
pc-CTA: Posterior circulation CT angiography
mTICI: Modified treatment in cerebral ischemia
GCS: Glasgow Coma Scale
MRI: Magnetic resonance imaging
NIHSS: The National Institutes of Health Stroke Scale

## References

[1] Amin-Hanjani S, Stapleton CJ, Du X, Rose-Finnell L, Pandey DK, Elkind MSV (2019). Hypoperfusion symptoms poorly predict hemodynamic compromise and stroke risk in vertebrobasilar disease. Stroke.

[2] Voetsch B, DeWitt LD, Pessin MS, Caplan LR (2004). Basilar artery occlusive disease in the new England medical center posterior circulation registry. Arch Neurol.

[3] Savitz SI, Caplan LR (2005). Vertebrobasilar disease. N Engl J Med.

[4] Merwick Á, Werring D (2014). Posterior circulation ischaemic stroke. BMJ.

[5] Goyal M, Menon BK, van Zwam WH, Dippel DW, Mitchell PJ, Demchuk AM (2016). Endovascular thrombectomy after large-vessel ischaemic stroke: a meta-analysis of individual patient data from five randomised trials. Lancet.

[6] Jovin TG, Li C, Wu L, Wu C, Chen J, Jiang C (2022). Trial of thrombectomy 6 to 24 hours after stroke due to basilar-artery occlusion. N Engl J Med.

[7] Tao C, Nogueira RG, Zhu Y, Sun J, Han H, Yuan G (2022). Trial of endovascular treatment of acute basilar-artery occlusion. N Engl J Med.

[8] Casetta I, Fainardi E, Saia V, Pracucci G, Padroni M, Renieri L (2020). Endovascular thrombectomy for acute ischemic stroke beyond 6 hours from onset: a real-world experience. Stroke.

[9] Hussein HM, Saleem MA, Qureshi AI (2018). Rates and predictors of futile recanalization in patients undergoing endovascular treatment in a multicenter clinical trial. Neuroradiology.

[10] Zaidat OO, Yoo AJ, Khatri P, Tomsick TA, von Kummer R, Saver JL (2013). Recommendations on angiographic revascularization grading standards for acute ischemic stroke: a consensus statement. Stroke.

[11] De Meyer SF, Denorme F, Langhauser F, Geuss E, Fluri F, Kleinschnitz C (2016). Thromboinflammation in stroke brain damage. Stroke.

[12] de Waha S, Patel MR, Granger CB, Ohman EM, Maehara A, Eitel I (2017). Relationship between microvascular obstruction and adverse events following primary percutaneous coronary intervention for ST-segment elevation myocardial infarction: an individual patient data pooled analysis from seven randomized trials. Eur Heart J.

[13] Rentzos A, Karlsson JE, Lundqvist C, Rosengren L, Hellström M, Wikholm G (2018). Endovascular treatment of acute ischemic stroke in the posterior circulation. Interv Neuroradiol.

[14] Da Ros V, Meschini A, Gandini R, Del Giudice C, Garaci F, Stanzione P (2016). Proposal for a vascular computed tomography-based grading system in posterior circulation stroke: a single-center experience. J Stroke Cerebrovasc Dis.

[15] Filep RC, Marginean L, Stoian A, Bajko Z (2021). Diagnostic and prognostic computed tomography imaging markers in basilar artery occlusion (Review). Exp Ther Med..

[16] Hui W, Wu C, Zhao W, Sun H, Hao J, Liang H (2020). Efficacy and safety of recanalization therapy for acute ischemic stroke with large vessel occlusion: a systematic review. Stroke.

[17] Zi W, Qiu Z, Wu D, Li F, Liu H, Liu W (2020). Assessment of endovascular treatment for acute basilar artery occlusion via a nationwide prospective registry. JAMA Neurol.

[18] Tao C, Qureshi AI, Yin Y, Li J, Li R, Xu P (2022). Endovascular treatment versus best medical management in acute basilar artery occlusion strokes: results from the ATTENTION multicenter registry. Circulation.

[19] Liu X, Dai Q, Ye R, Zi W, Liu Y, Wang H (2020). Endovascular treatment versus standard medical treatment for vertebrobasilar artery occlusion (BEST): an open-label, randomised controlled trial. Lancet Neurol.

[20] Langezaal LCM, van der Hoeven E, Mont'Alverne FJA, de Carvalho JJF, Lima FO, Dippel DWJ (2021). Endovascular therapy for stroke due to basilar-artery occlusion. N Engl J Med.

[21] Pirson FAV, Boodt N, Brouwer J, Bruggeman AAE, den Hartog SJ, Goldhoorn RB (2022). Endovascular treatment for posterior circulation stroke in routine clinical practice: results of the multicenter randomized clinical trial of endovascular treatment for acute ischemic stroke in the Netherlands registry. Stroke.

[22] Eskey CJ, Meyers PM, Nguyen TN, Ansari SA, Jayaraman M, McDougall CG (2018). Indications for the performance of intracranial endovascular neurointerventional procedures: a scientific statement from the American Heart Association. Circulation.

[23] Association NBoCM, Association CDGoNBoCM, Association NICGoNBoCM (2022). Chinese guidelines for early endovascular intervention of acute ischemic stroke 2022. Chin J Neurol.

[24] Huo X, Raynald, Gao F, Ma N, Mo D, Sun X (2020). Characteristic and prognosis of acute large vessel occlusion in anterior and posterior circulation after endovascular treatment: the ANGEL registry real world experience. J Thromb Thrombolysis.

[25] Weber R, Minnerup J, Nordmeyer H, Eyding J, Krogias C, Hadisurya J (2019). Thrombectomy in posterior circulation stroke: differences in procedures and outcome compared to anterior circulation stroke in the prospective multicentre REVASK registry. Eur J Neurol.

[26] Mokin M, Sonig A, Sivakanthan S, Ren Z, Elijovich L, Arthur A (2016). Clinical and procedural predictors of outcomes from the endovascular treatment of posterior circulation strokes. Stroke.

[27] Cooray C, Fekete K, Mikulik R, Lees KR, Wahlgren N, Ahmed N (2015). Threshold for NIH stroke scale in predicting vessel occlusion and functional outcome after stroke thrombolysis. Int J Stroke.

[28] Olivato S, Nizzoli S, Cavazzuti M, Casoni F, Nichelli PF, Zini A (2016). e-NIHSS: an expanded National Institutes of health stroke scale weighted for anterior and posterior circulation strokes. J Stroke Cerebrovasc Dis.

[29] Lian X, Xu D, Wu J, Lin M, Yin Q, Xu G (2013). Endovascular recanalisation therapy for prolonged basilar artery occlusion based on clinical-diffusion MRI mismatch. Clin Neurol Neurosurg.

[30] Werner MF, López-Rueda A, Zarco FX, Blasco J, San Román L, Amaro S (2019). Value of Posterior circulation ASPECTS and Pons-Midbrain Index on non-contrast CT and CT Angiography Source Images in patients with basilar artery occlusion recanalized after mechanical thrombectomy. Radiologia.

[31] Ritvonen J, Sairanen T, Silvennoinen H, Virtanen P, Salonen O, Lindsberg PJ (2021). Comatose with basilar artery occlusion: still odds of favorable outcome with recanalization therapy. Front Neurol.

[32] Luo G, Mo D, Tong X, Liebeskind DS, Song L, Ma N (2018). Factors associated with 90-day outcomes of patients with acute posterior circulation stroke treated by mechanical thrombectomy. World Neurosurg.

