# Supplementary material for: Influencing factors of futile recanalization after endovascular therapy for cerebral infarction with posterior circulation occlusion of large vessels: a retrospective study
Source: BMC Neurol. 2023 Mar 29;23:126. doi: 10.1186/s12883-023-03166-x (PMC10052860; doi:10.1186/s12883-023-03166-x)
Supplement: Supplementary file 1 — Additional file 1: Supplementary 1. The baseline characteristics of patients in our study. [file 12883_2023_3166_MOESM1_ESM.doc]

**Supplementary 1：The baseline characteristics of patients in our study.**

| Group | NO. | PC-CTA score | GCS score | Pontine -midbrain index(score) | Time from discovery to recanalization | Operation time | History of Atrial fibrillation | NIHSS score | Gastrointestinal bleeding |
| --- | --- | --- | --- | --- | --- | --- | --- | --- | --- |
| A |  |  |  |  |  |  |  |  |  |
|  | 1 | 1 | 6 | 2 | 435 | 72 | N | 18 | N |
|  | 2 | 1 | 5 | 2 | 330 | 130 | N | 36 | N |
|  | 3 | 5 | 9 | 5 | 840 | 120 | N | 21 | N |
|  | 4 | 2 | 6 | 2 | 285 | 45 | N | 26 | N |
|  | 5 | 4 | 6 | 0 | 671 | 100 | N | 12 | Y |
|  | 6 | 3 | 8 | 2 | / | 100 | N | 22 | N |
|  | 7 | 1 | 6 | 0 | 440 | 100 | Y | 22 | N |
|  | 8 | 2 | 13 | 2 | 480 | 90 | N | 10 | N |
|  | 9 | 1 | 6 | 1 | 435 | 72 | N | 16 | N |
|  | 10 | 1 | 14 | 3 | 540 | 40 | N | 18 | N |
|  | 11 | 2 | 15 | 2 | 830 | 130 | N | 2 | N |
|  | 12 | 1 | 14 | 2 | 255 | 112 | N | 8 | N |
|  | 13 | 1 | 15 | 1 | 570 | 120 | N | 8 | N |
|  | 14 | 1 | 15 | 2 | 255 | 102 | N | 0 | N |
|  | 15 | 3 | 6 | 2 | 680 | 130 | N | 13 | Y |
|  | 16 | 4 | 4 | 2 | 800 | 40 | N | 36 | N |
|  | 17 | 1 | 7 | 1 | 540 | 110 | N | 11 | N |
|  | 18 | 4 | 10 | 2 | 450 | 90 | N | 5 | N |
|  | 19 | 1 | 13 | 0 | 460 | 72 | N | 6 | N |
|  | 20 | 2 | 15 | 2 | 460 | 186 | N | 8 | N |
|  | 21 | 0 | 11 | 0 | 450 | 90 | N | 8 | N |
|  | 22 | 1 | 14 | 0 | 880 | 140 | N | 15 | N |
|  | 23 | 3 | 13 | 0 | / | 52 | Y | 12 | N |
|  | 24 | 2 | 14 | 2 | 222 | 57 | N | 10 | N |
|  | 25 | 1 | 15 | 0 | 565 | 112 | N | 14 | N |
|  | 26 | 2 | 6 | 0 | 480 | 40 | Y | 36 | Y |
|  | 27 | 1 | 15 | 1 | 720 | 140 | N | 2 | N |
|  | 28 | 3 | 5 | 0 | 570 | 105 | Y | 25 | N |
|  | 29 | 2 | 14 | 0 | 297 | 77 | Y | 2 | N |
|  | 30 | 1 | 10 | 0 | 580 | 105 | N | 12 | N |
|  | 31 | 1 | 14 | 0 | 395 | 102 | N | 6 | N |
|  | 32 | 6 | 6 | 0 | 537 | 180 | N | 13 | N |
|  | 33 | 3 | 15 | / | 560 | 155 | N | 2 | N |
|  | 34 | 4 | 8 | / | 520 | 60 | Y | 17 | N |
|  | 35 | 2 | 6 | / | 290 | 120 | N | 28 | N |
|  | 36 | 2 | 6 | / | 240 | 60 | N | 29 | N |
|  | 37 | 1 | 8 | / | 300 | 104 | Y | 18 | N |
|  | 38 | 4 | 7 | / | 1430 | 150 | N | 20 | N |
|  | 39 | 4 | 6 | / | 470 | 180 | N | 18 | N |
|  | 40 | 5 | 4 | / | 400 | 160 | N | 36 | N |
|  | 41 | 2 | 13 | / | 1200 | 177 | N | 13 | N |
|  | 42 | 4 | 13 | / | 570 | 140 | N | 16 | N |
|  | 43 | 2 | 10 | / | 535 | 105 | Y | 15 | N |
|  | 44 | 0 | 8 | / | 915 | 67 | N | 36 | N |
|  | 45 | 2 | 8 | / | / | 156 | N | 26 | N |
| B |  |  |  |  |  |  |  |  |  |
|  | 1 | 6 | 4 | 5 | 435 | 424 | N | 36 | Y |
|  | 2 | 0 | 8 | 4 | 1585 | 220 | Y | 20 | Y |
|  | 3 | 5 | 8 | 4 | 580 | 125 | N | 17 | N |
|  | 4 | 1 | 4 | 1 | 1570 | 140 | N | 36 | Y |
|  | 5 | 5 | 6 | 0 | 473 | 120 | N | 36 | Y |
|  | 6 | 1 | 5 | 0 | 3006 | 120 | N | 19 | Y |
|  | 7 | 4 | 12 | 3 | 1840 | 100 | N | 11 | N |
|  | 8 | 6 | 14 | 3 | 1119 | 60 | Y | 10 | N |
|  | 9 | 1 | 6 | 2 | 545 | 120 | N | 13 | Y |
|  | 10 | 3 | 7 | 4 | 718 | 250 | N | 36 | Y |
|  | 11 | 0 | 4 | 0 | 300 | 130 | N | 36 | N |
|  | 12 | 5 | 9 | 4 | 408 | 120 | N | 23 | Y |
|  | 13 | 4 | 2 | 2 | 3007 | 130 | N | 36 | N |
|  | 14 | 2 | 7 | 4 | 370 | 135 | N | 36 | Y |
|  | 15 | 6 | 3 | 2 | 302 | 220 | N | 36 | N |
|  | 16 | 1 | 10 | 3 | 550 | 115 | N | 11 | N |
|  | 17 | 5 | 6 | 2 | 150 | 55 | N | 36 | Y |
|  | 18 | 4 | 7 | 0 | 535 | 100 | N | 19 | N |
|  | 19 | 2 | 9 | 2 | 1002 | 115 | N | 12 | N |
|  | 20 | 4 | 14 | 4 | 615 | 120 | N | 9 | N |
|  | 21 | 1 | 14 | 0 | 1085 | 86 | N | 5 | N |
|  | 22 | 5 | 5 | 0 | 605 | 180 | N | 36 | N |
|  | 23 | 1 | 9 | / | 1447 | 110 | N | 7 | Y |
|  | 24 | 5 | 4 | / | 195 | 90 | N | 36 | N |
|  | 25 | 5 | 4 | / | 1260 | 68 | N | 36 | Y |
|  | 26 | 5 | 3 | / | 590 | 145 | N | 36 | N |
|  | 27 | 6 | 6 | / | 435 | 260 | N | 36 | Y |
|  | 28 | 5 | 3 | / | 240 | 160 | N | 36 | Y |
|  | 29 | 5 | 3 | / | 450 | 164 | N | 36 | Y |
|  | 30 | 6 | 5 | / | 1193 | 90 | N | 36 | N |
|  | 31 | 3 | 7 | / | 406 | 156 | N | 36 | Y |
|  | 32 | 6 | 5 | / | 293 | 101 | N | 36 | N |
|  | 33 | 1 | 6 | / | 735 | 200 | N | 36 | N |
|  | 34 | 5 | 8 | / | 1830 | 160 | N | 20 | N |
|  | 35 | 1 | 5 | / | 629 | 55 | N | 36 | N |
|  | 36 | 4 | 11 | / | 940 | 94 | N | 14 | N |
|  | 37 | 2 | 5 | / | 490 | 120 | N | 7 | N |
|  | 38 | 6 | 3 | / | 450 | 100 | N | 36 | N |
|  | 39 | 1 | 8 | / | 570 | 115 | N | 25 | N |
|  | 40 | 4 | 9 | / | 1620 | 110 | N | 19 | Y |
|  | 41 | 1 | 5 | / | 629 | 80 | N | 36 | N |
|  |  |  |  |  |  |  |  |  |  |

**Illustration:**

**Group A：the effective recanalization group**

**Group B：the ineffective recanalization group**

Due to some patients' incomplete brain MR and lack of imaging data, the pontine midbrain index score of these patients cannot be obtained; Y means the patient has atrial fibrillation, N means no atrial fibrillation.
